# Is High Definition MLC Dosimetrically Superior to Standard Definition MLC for SIB-SBRT for Carcinoma Prostate

**DOI:** 10.31557/APJCP.2019.20.12.3817

**Published:** 2019

**Authors:** Bijina T K, K M Ganesh, Pichandi A

**Affiliations:** 1 *Research and Development center, Bharathiar University, Coimbatore, *; 2 *Department of Radiation Oncology, Healthcare Global Enterprises, *; 3 *Department of Radiation Physics, Kidwai Memorial Institute of Oncology, Bangalore, India. *

**Keywords:** IMRT, VMAT, MLC, Prostate, SIB, SBRT

## Abstract

**Objective:**

The study was conducted to quantitatively evaluate the dosimetric effects of high definition (2.5 mm) and standard definition (5.0 mm) MLC on the quality of SBRT plans using SIB-IMRT and SIB-VMAT technique for carcinoma prostate and also to evaluate the dosimetric advantage of one technique over the other.

**Materials and Methods:**

Seventeen annonymized planning CT data sets were used to generate plans for both VMAT and IMRT techniques using 2.5 mm and 5.0 mm MLC.The prescription to the nodule was 45Gy in 5 fractions and to the prostate was 35Gy in 5 fractions.CI, GI, D_2%_, D_98%_, D_50%_ and V_95%_ for target; D_2%_, Dmean, V_80%_, V_20%_ for OAR’s; V_5%_ of the irradiated volume, and delivered MU’s were analyzed.An independent t-test was used to compare the plans. Patient specific QA for all plans were also performed and analyzed.

**Results:**

Minor difference in dosimetric indices was observed between 2.5mm and 5mm MLC VMAT plans, except D_2%_ (PTV35) and D_98%_ (GTV45) were better in 2.5mm MLC plans (p<0.05).D_2%_ and D_50%_ to GTV45 with p<0.009 and <0.03 respectively, and D_2%_ of bladder (p<0.03) were significantly lesser for VMAT plans compared to IMRT plans. GI was significantly better for 2.5mm MLC plans in both techniques (p<0.05). 2.5mm MLC plans on an average had 9.9% and 7% more MU for VMAT and IMRT plans respectively.

**Conclusion:**

All the plans provided adequate dose coverage to target. Highly gradient plans with better conformity were achieved with 2.5mm MLC and VMAT combination with an increase in MU delivered, compared to 5mm MLC plans.

## Introduction

Radiation therapy (RT) is one of the important modalities in treatment of cancers, which accounts for approximately 50% of all cancer patients. With the advancements in RT techniques when compared to the older conventional RT treatment techniques, intensity-modulated radiation therapy (IMRT) and volumetric-modulated arc therapy (VMAT), has resulted in an advantage of higher dose delivery to the cancer cells and minimized dose to the organs at risk (OAR) and healthy tissue. The higher dose delivery is made possible by the use of multileaf collimator (MLC), which generates a very steep dose gradient and non-uniform dose during treatment delivery with a constantly changing field shape in IMRT and with the further addition of a constantly moving gantry in VMAT (Woon et al., 2018).

The prostate is one of the most common tumor sites where dosimetric comparison between VMAT and IMRT is performed. Though with VMAT, shortened treatment time is a common finding, there are inconsistencies noted in the dosimetric outcome. Studies on dosimetric comparison in prostate-only or prostate with seminal vesicles found that one-arc VMAT provided equivalent or superior target coverage and OAR sparing compared with 5-f IMRT or 7-f IMRT (Ishii et al., 2013).

The resolution of the modulated photon beam intensities or fluence, in VMAT is determined by the MLC leaf width, just as in IMRT. Quite a few studies have investigated the relationship between MLC leaf width and the quality of IMRT plans and have shown that finer leaf widths can result in more conformal dose distributions for target volumes and less dose delivered to normal tissue when the target volumes were small (Rodal et al., 2010; Shang et al., 2013; Hong et al., 2014). Similarly, few studies have shown the dosimetric advantage of using MLCs with narrow leaf width (Lafond et al., 2013; Park et al., 2016). However, there are very little data on the comparison of the dosimetric effect of narrow width MLC on the quality of IMRT and VMAT plans with simultaneous integrated boost techniques in our study setting.

Consequently, this study was conducted to quantitatively evaluate the dosimetric effect of 2.5 mm and 5.0 mm MLC on the quality of simultaneous integrated boost (SIB) IMRT and VMAT stereotactic body radiotherapy (SBRT) plans for carcinoma prostate and also to evaluate the dosimetric advantage of one technique over the other.

## Materials and Methods

This is a retrospective study using 17 annonymized planning CT data sets previously treated with SBRT at our center. Plans were made for both VMAT and IMRT techniques using 2.5 mm and 5.0 mm MLC.

The entire prostate gland was contoured as CTV with 0.3cm margin given around CTV to create PTV35. The dominant nodule within the prostate gland was contoured as GTV45 with no margins. Organs at risks (OARs) such as the urinary bladder, rectum, penile bulb urethra, small bowel and bilateral femoral heads were delineated.

*Treatment planning*

The CT data and RT structures were exported to the planning system and the treatment plans were generated with Eclipse version 13.6 (Varian Medical Systems, Palo Alto, CA) TPS for which QA is done periodically. For IMRT plan optimization, Dose Volume Optimizer and for VMAT, Progressive Resolution Optimizer were used. The dose calculation was performed using Anisotropic Analytical Algorithm. The prescribed dose to PTV35 was 35 Gy in 5 fractions with simultaneous integrated boost to GTV45 with 45Gy in 5 fractions. The parameters such as the isocenter location, the number of fields, the MLC margin, the gantry, collimator, and couch angles for each beam and the dose constraints were constant for both MLC plans. The set treatment planning dosimetry goals were enumerated in Table 1. No limit on target dose heterogeneity was specified, but effort was made to limit the maximum dose (Dmax) to GTV <120%. The IMRT plans consisted of single-isocenter, coplanar, and 9 equidistant fields delivered in dynamic MLC mode. The VMAT plans were implemented with a single-isocenter 2-full arc, and without couch rotation. The collimator angles were set to 30° and 330° for each arc. The gantry angle range of each arc was 179.9°-181.1°. Thus, 68 treatment plans were generated for two MLC leaf widths (2.5 mm and 5.0 mm), and two types of planning techniques (VMAT and IMRT). All the plans were normalized to cover 95% of GTV45 and PTV35 to their respective prescription dose. The dosimetric outcomes were compared for both MLC’s and between the treatment techniques.

*Evaluation parameters*

The evaluation parameters included dose received by 98% of both GTV45 and PTV35 (D_98%_), D_2%_, D_50%_, the percentage volume of target receiving at least 95% of the prescription dose (V_95%_), Conformity Index (CI), and Gradient Index (GI). The definition of each index is summarized below.

CI: The ratio used to evaluate the quality of fit of the target volume to the prescription isodose volume. A score of 1 represents a perfectly conformal plan.

CI = (TV_PIV_/PIV) x (TV_PIV_/TV)

Wherein, VPIV represents volume of PTV receiving the prescription dose; PIVrepresents prescription isodose volume and TV represents target volume (Van’t Riet et al., 1997). CI is calculated for both PTV35 and GTV45.

GI: The index that represents the degree of dose drop-off outside the target volume. A smaller value of GI indicates a better degree of dose drop-off outside the target volume.

GI = PIV_50_/PIV

Where PIV_50_ is the volume receiving at least 50% dose of the prescription dose; PIV is the prescription isodose volume (Paddick, Lippitz 2006).

The irradiated volume receiving more than 5% of the prescription dose (V_5%_) is recorded for all the plans. The mean dose, D2%and the dose volumes V_80%_ and V_20% _were analyzed for bladder and rectum. D_2%_ and mean dose were compared for bilateral femoral heads. Plan efficiency was determined using the treatment delivery parameters such as MU and estimated treatment time per fraction.

*Statistical analysis*

All the data were entered into MS Excel sheet. An independent t-test was used to analyze and compare the dosimetric indices between IMRT and VMAT plans according to the MLC leaf width. The statistical analysis was conducted using SPSS version 16.0 and a p value of <0.05 was considered statistically significant.

## Results

**Figure 1:**
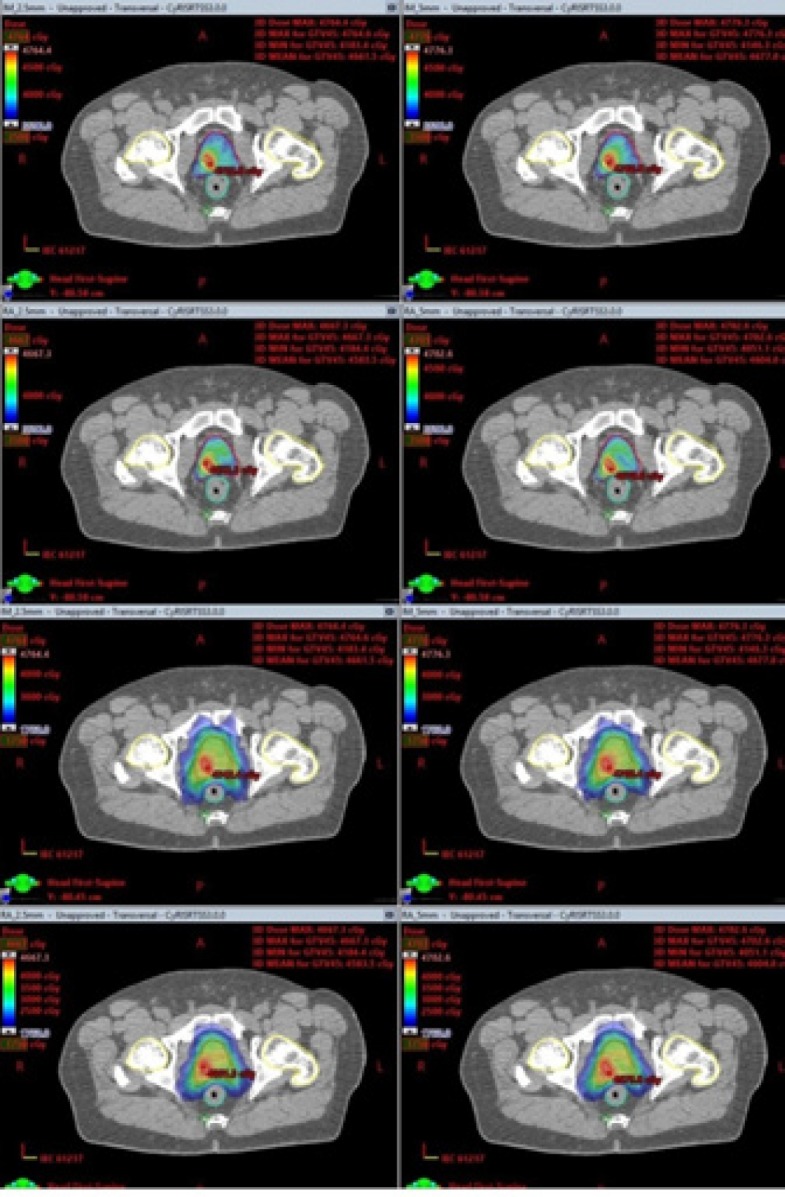
Isodose Comparison of One Representative Patient

Seventeen patients were included in this study. The median volume of GTV45 and PTV35 was 5.2cm^3^ and 70.8 cm^3^ respectively. The DVH evaluation parameters averaged over all patients were shown in Table 2, 3 and 4. A comparison of dose distributions for both MLC and treatment technique combination of one representative patient was shown in Figure 1.

*Dosimetric analysis of 5.0mm v/s2.5mm MLC in VMAT*

The minimum dose received by 2% of the PTV35was noted to be significantly higher in 5.0mm MLC (5.0mm v/s 2.5mm: 43.22±0.58 Gy v/s 42.68±0.49 Gy) (p<0.05). The minimum dose received by 98% of the PTV35 (5.0mm v/s 2.5mm: 34.07±0.35 Gy v/s 34.29±0.24 Gy) and GTV45was significantly higher in 2.5mm MLC (5.0mm v/s 2.5mm: 44.44±0.14 Gy v/s 44.60±0.16 Gy) (p<0.05). The CIGTV45 and CIPTV35 did not vary significantly with the width of MLC (p>0.05) as shown in Table 3. Dmean, D_2%_, V_80%_ and V_20% _of bladder, rectum, left and right femur were not significant in either of the MLC widths (p>0.05) as shown in Table 4. No statistically significant difference was observed with respect to MU between 5mm and 2.5mm MLC plans.

*Dosimetric analysis of 5.0mm v/s 2.5mm MLC in IMRT*

Among the dosimetric indices and DVH parameters viz., minimum dose received by 2%, 50% and 98% of the PTV35and GTV35, CI, GI and V_5%_to irradiated volume, none of the indices varied significantly with the width of MLC (p>0.05) but for the GI which was better in 2.5mm MLC for IMRT wherein it was significantly lower (5.0mm v/s 2.5mm: 4.38±0.34 v/s 4.04±0.38) (p<0.05). Damon and D_2% _and V_80%_ and V_20%_ of bladder, rectum, left and right femur were not significant in either of the MLC widths (p>0.05) as shown in Table 4. In IMRT technique, 2.5mm MLC plans produced more MU compared to 5mm MLC plans which is not statistically significant (p>0.05).

*Dosimetric analysis of VMAT v/s IMRT*

The minimum dose received by 50% (VMAT v/s IMRT: 34.29±0.24 Gy v/s 33.93±0.47 Gy) and 98% (VMAT v/s IMRT: 38.51±0.75 Gy v/s 37.47±0.59 Gy) of the PTV35 was significantly higher in VMAT for 2.5mm and 5.0mm MLC respectively. D_2%_ of GTV45 was significantly lesser in VMAT for 2.5mm MLC (VMAT v/s IMRT: 47.67±0.45 Gy v/s 48.31±0.67 Gy) and for D50% both 2.5mm (VMAT v/s IMRT: 46.44±0.25 Gy v/s 46.80±0.49 Gy) and 5.0mm (VMAT v/s IMRT: 46.55±0.24 Gy v/s 46.86±0.44 Gy) MLC respectively (p<0.05). The CIPTV35 and CIGTV45 was no different either between the techniques except in 2.5mm MLC which was significantly better in VMAT compared to IMRT (VMAT v/s IMRT: 0.75±0.08 v/s 0.67±0.09, 0.89±0.03 v/s 0.83±0.03) respectively (p<0.05). The GI was significantly better in VMAT when compared to the IMRT technique for 2.5mm MLC (VMAT v/s IMRT: 3.81±0.24 v/s 4.04±0.38). Damon and D_2%_and V_80% _and V_20%_ of bladder, rectum, left and right femur were not significant in either of the techniques except for D_2% _for bladder in IMRT technique in 2.5mm MLC (VMAT v/s IMRT: 25.62±5.47 Gy v/s 29.66±3.67 Gy) which was significantly lesser in VMAT technique (p<0.05) as shown in Table 4. 

For each patient, four QA plans were delivered in PTW Octavius 729 array detector. All plans yielded equivalent results and passed our criteria of gamma index with ≥ 95% with 3%, 3mm distance to agreement as shown in Table 5. 

**Table 1:** Dose Constraints of Target and OAR’s

Structure	Planning goals
Targets	
GTV45	V_45Gy_ ≥95%
PTV35	V_35Gy_ ≥95%
OAR’s	
Rectum	V_50%_(22.5Gy)< 50%
	V_80%_(36Gy)< 20%
	V_100%_(45Gy) < 5%
Bladder	V_50%_ (22.5Gy)< 40%
	V_100%_ (45Gy)<5%
Urethra	Max point dose should not exceed 110%
Femoral heads	V_40%_(18Gy) < 5%

*OAR, Organ At Risk; V45Gy, Volume receiving 45 Gy; V35Gy, Volume receiving 35 Gy; V_50%_, Volume receiving 50% of prescription dose; V_80%_, Volume receiving 80% of prescription dose; V_100%_, Volume receiving 100% of prescription dose; V_40%_, Volume receiving 40% of the prescription dose

**Table 2:** DVH Parameters among 5.0mm vs2.5mm MLC and VMAT vs IMRT

Variables	Treatment Techniques	MLCs leaf width		p-value between MLCs
		5.0mm	2.5mm	
PTV35				
D_2%_ (Gy)	VMAT	43.22±0.58	42.68±0.49	0.02*
	IMRT	42.97±1.09	42.64±2.64	0.68
	*P-value* between techniques	0.48	0.95	
	VMAT	34.07±0.35	34.29±0.24	0.07*
D_98%_ (Gy)	IMRT	34.02±0.36	33.93±0.47	0.57
	*P-value* between techniques	0.73	0.02*	
	VMAT	38.51±0.75	38.15±0.56	0.18
D_50%_ (Gy)	IMRT	37.47±0.59	37.79±0.56	0.16
	*P-value* between techniques	0.001*	0.12	
	VMAT	99.46±0.29	99.62±0.15	0.09
V_95%_ (%)	IMRT	99.38±0.36	99.41±0.43	0.88
	*P-value* between techniques	0.58	0.11	
GTV45				
	VMAT	47.88±0.57	47.67±0.45	0.31
D_2%_ (Gy)	IMRT	48.14±0.52	48.31±0.67	0.48
	*P-value* between techniques	0.25	0.009*	
	VMAT	44.44±0.14	44.60±0.16	0.01*
D_98%_ (Gy)	IMRT	44.39±0.21	44.65±0.79	0.29
	*P-value* between techniques	0.52	0.86	
	VMAT	46.55±0.24	46.44±0.25	0.26
D_50%_ (Gy)	IMRT	46.86±0.44	46.80±0.49	0.75
	*P-value* between techniques	0.03*	0.028*	
	VMAT	99.89±0.10	99.93±0.09	0.36
V_95%_ (%)	IMRT	99.83±0.21	99.87±0.21	0.67
	*P-value* between techniques	0.31	0.32	

* indicates differences to be statistically significant (p<0.05); Values represent Mean±SD; D_2%_ , - Dose to 2% volume, D_98%_- Dose to 98% volume, D_50%_- Dose to 50% volume,V_95%_ - Volume receiving 95% of prescription dose

**Table 3:** Dosimetric Indices among 5.0mm vs 2.5mm MLC and VMAT vs IMRT

Variables	Treatment Techniques	MLCs leaf width		p-value between MLCs
		5.0mm	2.5mm	
CI_GTV45_	VMAT	0.70±0.09	0.75±0.08	0.13
	IMRT	0.69±0.09	0.67±0.09	0.45
	*P-value* between techniques	0.88	0.02*	
CI_PTV35_	VMAT	0.85±0.07	0.89±0.03	0.29
	IMRT	0.82±0.03	0.82±0.03	0.85
	*P-value* between techniques	0.45	0.009*	
GI	VMAT	3.81±0.30	3.81±0.24	0.98
	IMRT	4.38±0.34	4.04±0.38	0.02*
	*P-value* between techniques	0.09	<0.0001*	
MUs	VMAT	2806.23±429.50	3085.32±650.25	0.21
	IMRT	2739.31±318.09	2930.03±350.49	0.16
	*P-value* between techniques	0.66	0.46	
V_5Gy_ (cc)	VMAT	2760.68±736.76	3056.49±835.80	0.35
	IMRT	2458.29±602.43	2743.91±698.32	0.28
	*P-value* between techniques	0.26	0.31	

* indicates differences to be statistically significant (p<0.05); Values represent Mean±SD; CIGTV45- Conformity Index (GTV45); CIPTV35- Conformity Index (PTV35); GI- Gradient Index; MU- Monitor units; V5Gy-Volume receiving 5Gy

**Table 4:** Comparison of Dose Received by OAR’s

Variables	Treatment Techniques	MLC leaf width		p-value between MLCs
		5.0mm	2.5mm	
Bladder				
V_80%_§	VMAT	0.07 (0-0.67)	0.02 (0-0.59)	0.54
[Median (Range)]	IMRT	0.01 (0-0.92)	0.01 (0-0.7)	0.58
	*P-value* between techniques	0.96	0.72	
V_20%_	VMAT	35.69±12.22	36.68±12.42	0.84
	IMRT	38.42±11.03	44.22±11.64	0.21
	*P-value* between techniques	0.56	0.12	
Dmean (Gy)	VMAT	8.17±2.26	8.27±2.21	0.91
	IMRT	8.69±1.98	9.88±2.40	0.18
	*P-value* between techniques	0.53	0.09	
D_2%_ (Gy)	VMAT	25.86±5.67	25.62±5.47	0.91
	IMRT	28.94±3.72	29.66±3.67	0.63
	*P-value* between techniques	0.11	0.03*	
Rectum				
V_80%_§	VMAT	0.15 (0-0.97)	0.15 (0-0.99)	0.72
[Median (Range)]	IMRT	0.14 (0-1.02)	0.13 (0-1.34)	0.8
	*P-value* between techniques	0.58	0.42	
	VMAT	48.64±13.28	50.72±14.10	0.7
V_20%_	IMRT	54.35±15.62	58.72±16.34	0.49
	*P-value* between techniques	0.33	0.19	
D_mean_ (Gy)	VMAT	9.85±2.28	9.77±2.22	0.93
	IMRT	10.38±2.26	11.43±2.29	0.25
	*P-value* between techniques	0.55	0.07	
D_2%_ (Gy)	VMAT	28.19±3.27	27.97±3.29	0.87
	IMRT	29.56±3.46	30.81±4.09	0.41
	*P-value* between techniques	0.31	0.06	
Lt.Femur				
D_mean_ (Gy)	VMAT	7.88 ±1.98	8.24±2.22	0.67
	IMRT	7.77±2.03	7.46±2.15	0.71
	*P-value* between techniques	0.89	0.37	
D_2%_ (Gy)	VMAT	12.46±1.85	13.18±5.04	0.63
	IMRT	13.54±1.49	13.45±2.04	0.89
	*P-value* between techniques	0.12	0.86	
Rt. Femur				
D_mean_ (Gy)	VMAT	7.89±1.92	7.98±1.76	0.89
	IMRT	8.03±2.09	7.75±2.05	0.73
	*P-value* between techniques	0.86	0.75	
D_2%_ (Gy)	VMAT	12.06±2.11	13.41±4.85	0.37
	IMRT	13.37±1.27	13.28±1.51	0.86
	*P-value* between techniques	0.07	0.93	

* indicates differences to be statistically significant (P<0.05); §-Mann Whitney U test applied; V_80%_- Volume receiving 80% of prescription dose, V_20%_- Volume receiving 20% of prescription dose; V_20%_- Volume receiving 20% of prescription dose, D_2%_- Dose to 2% volume

**Table 5:** Gamma Analysis Percentage Passing Rate (DD 3%, DTA 3 mm)

Patient No.	IMRT		VMAT	
	2.5 mm MLC	5 mm MLC	2.5 mm MLC	5 mm MLC
1	99.5	99.7	99.2	99.3
2	98.7	99.0	98.2	98.9
3	99.1	99.1	99.3	99.2
4	97.5	98.4	98.6	99.0
5	99.5	100	99.4	99.5
6	96.4	97.1	98.5	98.8
7	97.4	97.8	97.8	98.3
8	97.9	97.8	97.3	97.8
9	98.2	98.1	99.4	99.5
10	99.2	99.8	99.5	99.0
11	100.0	100.0	100.0	100.0
12	95.8	96.5	96.8	98.3
13	98.3	99.0	99.5	99.4
14	95.7	96.3	96.9	97.2
15	99.9	100.0	100.0	100.0
16	96.3	97.5	97.8	97.5
17	98.9	98.5	99.5	100.0

DD, Dose Difference; DTA, Distance to Agreement; IMRT, Intensity Modulated Radiotherapy; VMAT, Volumetric Modulated Arc Therapy; MLC, Multileaf collimator

## Discussion

In view of developments in MLCs along with the improvements in radiotherapy planning techniques and delivery methods, radiosurgery has been extensively used and dosimetric superiority and clinical effectiveness have been elicited in the literatures (Hong et al., 2014). However, there has paucity of research on the effects of MLC leaf width on volumetric-modulated arc therapy (VMAT) and also in IMRT in the current study setting. Hence we have compared the dosimetric indices in VMAT and IMRT and also the superiority of one technique over the other. The treatment plans were successful in satisfying the protocol-defined goals in all generated plans. The PTV and GTV coverage was excellent, with 95% of PTV35 and GTV45received the prescription dose in all the plans. All plans used in this study also met the OAR constraints.

The minimum doses received by 98% of the PTV and GTV were significantly higher in 2.5mm MLC VMAT and similar results with respect to dosimetric indices except for the significance has been observed in few studies (Chaeet al., 2014; Serna et al., 2015; Zhang et al., 2018). The minimum doses received by 2% of the PTV were noted to be significantly higher in 5.0mm MLC. The impact of MLC of 5-mm and 10-mm leaf width on VMAT in the case of prostate was found with increased OARs sparing for thinner MLCs (Kesterenet al., 2012). 

Jacob et al., (2010) demonstrated only small dosimetric effects of leaf widths between 2.5 mm and 5 mm in regard to target coverage. There was no significant difference in sparing of OAR’s between the IMRT plans with different leaf widths. The mean GI was significantly better in 2.5mm MLC in a study by Chae et al., (2014), and it was correlating to the current study finding where in it was significantly better in 2.5mm.

A study by Quan et al., (2012) revealed that IMRT plan quality was similar or superior to that of VMAT when the number of beams in IMRT was increased to a certain number between 12 to 24.Many studies mention VMAT to be better in target volume coverage (Palma et al., 2008; Kristoffersen et al., 2009; Hardcastle et al., 2011). When compared VMAT with IMRT plans for ten prostate patients, better rectal, bladder and femoral head sparing or normal tissue sparing was observed with the VMAT plans over the IMRT and 3DCRT plans (Palma et al., 2008; Kristoffersenet al., 2009). Similarly, with better gradient index in VMAT, the D2 dose delivery for bladder in 2.5mm MLC was significantly less compared to IMRT and it was achieved in spite of better conformity in IMRT (Palma et al., 2008; Kristoffersenet al., 2009; Hardcastle et al., 2011).

Though statistically insignificant difference in MU delivered was observed between 5mm and 2.5mm leaf width for both techniques in our study, leakage radiation is one of the major components that would directly correlate with increase in MU delivered. Many literatures have reported that leakage radiation would contribute to the integral dose to peripheral normal tissue. This may lead to increased risk of secondary malignancy (Sakthivel et al., 2017).

Highly conformal dose distribution with different dose levels within the primary tumor can be planned and delivered with the help of modern radiotherapy techniques. The combination of two MLC leaf widths with two different techniques was explored in this study. All the plans provided adequate dose coverage to target. Highly gradient plans with better conformity were achieved with 2.5mm MLC and VMAT combination with an increase in MU delivered, compared to 5mm MLC plans. The 2.5mm MLC in combination with VMAT technique resulted in dosimetric benefits in the treatment of prostate SIB SBRT.

## Conflict of interest

Authors have declared no conflict of interest.
